# Tuberculosis in Goats and Sheep in Afar Pastoral Region of Ethiopia and Isolation of *Mycobacterium tuberculosis* from Goat

**DOI:** 10.1155/2012/869146

**Published:** 2012-07-17

**Authors:** Gezahegne Mamo Kassa, Fekadu Abebe, Yalelet Worku, Mengistu Legesse, Girmay Medhin, Gunnar Bjune, Gobena Ameni

**Affiliations:** ^1^Aklilu Lemma Institute of Pathobiology, Addis Ababa University, P.O. Box 1176, Addis Ababa, Ethiopia; ^2^Faculty of Veterinary Medicine, Addis Ababa University, P.O. Box 34, Debre Zeit, Ethiopia; ^3^Faculty of Medicine, Institute of Health and Society, Department of Community Medicine, International Community Health, University of Oslo, P.O. Box 1130, Blindern N-0318 Oslo, Norway; ^4^Afar Pastoral Agricultural Bureau, Afar Pastoral Region, P.O. Box 73, Semera, Ethiopia

## Abstract

A cross sectional study was conducted on 2231 small ruminants in four districts of the Afar Pastoral Region of Ethiopia to investigate the epidemiology of tuberculosis in goats and sheep using comparative intradermal tuberculin skin test, postmortem examination, mycobacteriological culture and molecular typing methods. The overall animal prevalence of TB in small ruminants was 0.5% (95% CI: 0.2%–0.7%) at ≥4 mm and 3.8% (95% CI: 3%–4.7%) at cutoff ≥2 mm. The herd prevalence was 20% (95% CI: 12–28%) and 47% (95% CI: 37–56%) at ≥4 mm and ≥2 mm cut-off points, respectively. The overall animal prevalence of Mycobacterium avium complex infection was 2.8% (95% CI: 2.1–3.5%) and 6.8% (95% CI: 5.8–7.9%) at ≥4 mm and ≥2 mm cut-off points, respectively. Mycobacteriological culture and molecular characterization of isolates from tissue lesions of tuberculin reactor goats resulted in isolation of *Mycobacterium tuberculosis* (SIT149) and non-tuberculosis mycobacteria as causative agents of tuberculosis and tuberculosis-like diseases in goats, respectively. The isolation of *Mycobacterium tuberculosis* in goat suggests a potential transmission of the causative agent from human and warrants further investigation in the role of small ruminants in epidemiology of human tuberculosis in the region.

## 1. Background 

Ethiopia has one of the largest resources of goats and sheep among African countries, with an estimated number of 21.9 million goats and 25.9 million sheep [1]. Goats and sheep contribute significantly to the economy and food security of the poor farmers in the country. About 73% of the national goat population and 25% of the sheep population are found in the lowland pastoral areas of the country [2]. In pastoralist area, goats and sheep are mainly utilized for milk and meat production and generate income to the owner. In spite of the large population and potential use of small ruminants, the production system is affected by feed shortage, poor genetic makeup of the animals, and wide spread occurrence of livestock diseases such as tuberculosis which has both economic and public health significance to the communities. 

TB in goat and sheep is caused by members of *Mycobacterium tuberculosis* complex predominantly by *Mycobacterium bovis* and *Mycobacterium caprae *[3–14] and few caused by *Mycobacterium tuberculosis* [15, 16]. Epidemiological studies indicated that tuberculosis in goat and sheep has a wide global distribution and has been reported in various countries of the world including New Zealand, Sudan, Spain, Nigeria, the United Kingdom, Italy, Algeria, Ethiopia [3–17]. In Ethiopia, bovine TB has been known to be endemic in cattle; however, the status of TB in goats and sheep has not been well studied in spite of their close contact with cattle. Few studies carried out so far in central highland Ethiopia indicated the existence of TB in small ruminants with low level of prevalence (4.2%) based on abattoir examination results [14] and 3.1% using single intradermal tuberculin test [17]. In lowland pastoral area where the large population of goats and sheep exists, the status of the disease is unknown. 

Livestock in pastoralist region is major source of food and income, in addition, possession of livestock provides a measure of social status in the pastoral communities. In pastoralist communities of Ethiopia including the Afar, pastoralists' habit of consumption of raw animal product particularly milk is common and the pastoralists have close physical contact with their animals. Afar pastoralists consume both goat and sheep milk very commonly, and to protect these small ruminants from predators, the pastoralists keep these animals in very close proximity to their houses. These conditions are potential risk factors for transmission of zoonotic diseases such as TB of animal origin to human or vice versa. Goats and sheep have also common watering and grazing points with cattle that might favor the transmission of mycobacterial species among these domestic animals. Previous studies in cattle and camel of pastoral regions indicated the endemic nature of TB in the regions [18–22]. Therefore, the present study was designed to investigate the epidemiology of TB in goats and sheep and characterizes the causative agents in the Afar Pastoral Region of Ethiopia. 

## 2. Material and Methods

### 2.1. Study Area

The study was conducted in four districts, namely, Amibara, Dubti, Afambo, and Chifra districts of Afar Pastoral Region. The Afar Pastoral Region is located in northeast of Ethiopia between 39°34′ and 42°28′ E longitude and 8°49′ and 14°30′ N latitude. The region shares common international boundaries with Eritrea in the northeast and Djibouti in the east, and it is characterized by an arid and semiarid climate with low and erratic rainfall [23]. 

In the Afar Region, there are about 4,268,000 goats and 2,464,000 sheep which are managed under pastoral and agropastoral production system [24]. Afar pastoralists own different species of domestic animals, and these animals share common watering points and grazing sites. Small ruminants usually graze/browse near their villages, while cattle and camel might travel a long distance in search of grass and browsing trees. The watering points of small ruminants are commonly shared with cattle and camel creating a close interspecies interaction among these domestic animals, and this might increases the risk of transmission of mycobacteria from cattle or camels to small ruminants or vice versa. 

### 2.2. Study Design

A cross-sectional study was conducted in the four districts of the Afar Pastoral Region and a total of 14 subdistricts and 21 villages were included in the study based on the inclusion criteria (accessibility, security, and willingness of the pastoralists to participate in the research). All villages in each subdistrict were included after obtaining the elder clan leaders' consent to participate in the study. In this study, goat and sheep kept by an owner and his close relatives in which case if the animals share common grazing sites, watering points, and night shelter, they were considered as a herd to calculate the herd prevalence. A total of 103 flocks (herds) of small ruminants were tested by CIDT test. 

### 2.3. Study Animals

For the CIDT test, small ruminants above the age of six months having no clinical symptom of any disease were included. Study animal-related information on each tested sheep and goat (such as sex, age, body condition score, lactation and reproductive status, and parity number) was collected and recorded at the time of the test. Each animal was dewormed with antihelmintic drug after testing. A total of 2231 small ruminants (1884 goat and 347 sheep) were tested using CIDT. 

### 2.4. Comparative Intradermal Tuberculin Skin Test (CIDT)

CIDT test was carried out by injecting both bovine purified protein derivative (PPD) and avian PPD (observe bovine and avian tuberculin, AsureQuality Company, Mt. Wellington, Auckland, New Zealand). Two sites on the skin of the mid-neck of the animal, 12 cm apart, were shaved, and skin thickness was measured with a caliper. One site was injected with an aliquot of 0.1 mL of 2,500-IU/mL bovine PPD into the dermis, and the other was similarly injected with 0.1 mL of 2,500-IU/mL avian PPD. After 72 h, the skin thickness at the injection site was measured and recorded. Results were interpreted according to the recommendations of the Office International des Epizooties [25] at ≥4 mm cutoff and also at ≥2 mm cutoff [26]. Thus, at cutoff ≥4 mm, if the increase in skin thickness at the injection site for bovine PPD (PPD-B) was greater than the increase in skin thickness at the injection site for avian PPD (PPD-A) and PPD-B minus PPD-A was less than 2 mm, between 2 and 4 mm, or 4 mm and above, the animal was classified as negative, doubtful, or positive reactor based on CIDT test, respectively. At cutoff ≥2 mm, if the difference between PPD-B and PPD-A was greater or equal to 2 mm, the animal was considered as positive, while if the difference is less than 2 mm, the animal was considered as negative. When the change in skin thickness was greater at PPD-A injection site, the animal was considered positive for mycobacteria species other than *Mycobacterium tuberculosis* complex. A flock (herd) was considered as positive if it had at least one tuberculin reactor animal. 

### 2.5. Body Condition Scoring

The body condition scoring for goat and sheep was carried out using the guidelines established by Langston University and ESGIP guidelines for body condition scoring [27, 28]. Accordingly, on the basis of observation of anatomical parts such as vertebral column, ribs, and spines, the study animals were classified as lean (score 1 to 2), medium (3 to 4), or fat (greater than 5). 

Age determination was carried out based on the dentition according to Vatta and his coworkers [29] and adopted ESGPIP guideline for estimation of age of sheep and goat [30]. 

### 2.6. Postmortem  Examination

 Tissues with suspicious lesions from five slaughtered tuberculin reactor goats were collected aseptically from the lung lobes (left apical, left diaphragmatic, right apical, right cardiac, right diaphragmatic, and right accessory), lymph nodes of the head (retropharyngeal and mandibular), lymph nodes of lungs (mediastinal and bronchial), and mesenteric lymph nodes. Data were collected on the presence, size, and distribution of visible lesions in each carcass. Samples from tissues containing visible lesions were collected and placed into sterile universal bottles containing 5 mL of 0.9% saline solution(pH 7.2)and kept at −20°C at Semera Regional Animal Health Laboratory until they were transported to ALIPB laboratory under cold chain for isolation of the causative agents.

### 2.7. Isolation of Mycobacteria from Tissue Samples

Isolation of mycobacteria from tissues was done in accordance with OIE protocols [31]. Briefly, the specimens were sectioned into pieces using sterile blades and then homogenized by pestle and mortar. The homogenate was decontaminated by adding an equal volume of 4% NaOH followed by centrifugation at 1000 g for 15 minutes. The supernatant was discarded, while the sediment was neutralized by 1% (0.1N) HCl using phenol red as an indicator. Neutralization was achieved when the colour of the solution changed from purple to yellow. Thereafter, 0.1 mL of suspension from each sample was spread onto a slope of Löwenstein-Jensen (LJ) medium. Duplicates of LJ media were used; one enriched with sodium pyruvate, while the other was enriched with glycerol. Cultures were incubated at 37°C in a slant position for one week and in upright position for 11 weeks with weekly observation for mycobacterial growth. Whenever, colonies were seen, subculturing and Ziehl-Neelsen staining were performed to confirm the presence of acid fast bacilli. Positive colonies were preserved with freezing media, and some portions of the colonies were heat-killed in water bath at 80°C for 45 minutes. The frozen and heat killed isolates were stored at −20°C for future mycobacteriology and further molecular typing analysis.

### 2.8. Molecular Characterization of Mycobacterial Isolates

Mycobacterium genus typing was conducted as described previously [32], and spoligotyping of *Mycobacterium tuberculosis* complex isolate from goat was performed as previously described by Kamerbeek and coauthors [33]. Both methods were described in detail in previous publication [22].

### 2.9. Data Management and Analysis

Data were classified, filtered, coded using EpiData software and Microsoft Excel sheet, and was transferred and analyzed using STATA version 11 (Stata Corp., Collage station, TX). Pearson chi-square was used to evaluate the statistical significance of the associations of different categorical variables with skin test results. Bivariate and multivariable logistic regression analyses were performed to quantify crude and adjusted effects of prespecified risk factors on tuberculin reactivity. *P* values less than 5% were considered statistically significant. In cases of estimating the effect of different risk factors in terms of OR with corresponding 95% confidence interval, statistically significance was assumed if the confidence interval did not include one among its values. 

## 3. Results

### 3.1. Animal Prevalence

On the basis of CIDT test, the animal prevalence of TB was 0.5% (10/2231) at a cutoff ≥4 mm and 3.8% (86/2231) at a cutoff ≥ 2 mm. At ≥2 mm cut-off point, there were significant differences in proportions of reactors among the four districts (*χ* ^2^ = 26.385, *P* = 0.000), between sheep and goat (*χ* ^2^= 6.46, *P *= 0.011) and between pregnant and nonpregnant females (*χ* ^2^= 5.342, *P* = 0.021) (Table 1). Multivariable logistic regression analysis at ≥2 mm cut-off point showed that older small ruminants (5 years and above) had 13 times the odds of being tuberculin reactors compared with age category less than 2 years old (adjusted OR = 13.79; CI: 2.22–85.55). Female small ruminants with parity number greater than 4 had 0.05 odds of being bovine tuberculin positivity in relative to those with less than 2 parity numbers (adjusted OR = 0.05; CI: 0.01–0.31) (Table 2). At ≥4 mm cut-off points, there was no statistical significance difference in the proportion of bovine tuberculin positivity between groups in relation to the different variables considered. 

### 3.2. Herd Prevalence

The herd prevalence was 20% (95% CI: 12–28%) and 47% (95% CI: 37–56%) at ≥4 mm and ≥2 mm cut-off points, respectively. In multivariable logistic regression analysis, no significant association was found in herd positivity between groups in relation to district of origin, herd size category, and production system at ≥2 mm cut-off point (Table 3).

### 3.3. Prevalence of *Mycobacterium Avium* Complex Infection

According to the CIDT test result of the avian tuberculin skin reaction, the overall animal prevalence of *Mycobacterium avium* complex infection was 2.8% (95% CI: 2.1–3.5%) and 6.8% (95% CI: 5.8–7.9%) at ≥4 mm and ≥2 mm cut-off points, respectively. 

### 3.4. Postmortem Lesions in Tuberculin Reactor Goats

**Figure 1:**
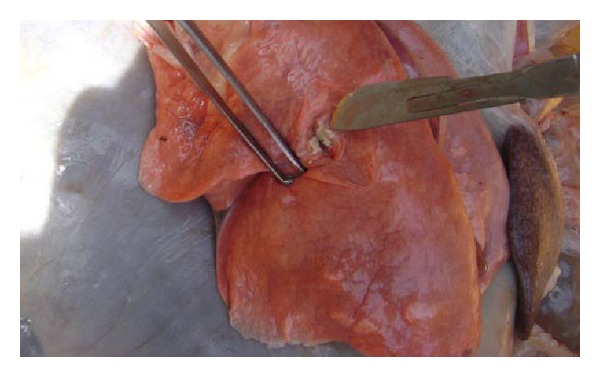
Tuberculous lesion from goat lung caused by *Mycobacterium tuberculosis. *

Five goats which were positive to bovine TB in CIDT test at cutoff ≥ 4 mm were purchased, slaughtered, and investigated for gross tuberculous lesions. Tuberculous lesions were detected in different organs (left diaphragmatic lung, retropharyngeal lymph node, parotid lymph node, right bronchial lymph node, mesenteric lymph node, intestinal wall, and mesentery). Two of them had partially disseminated TB lesions which involved lung, intestine and the lymph nodes of thoracic and abdominal cavities. Upon incision of the lung, lesions showed a yellowish caseous material indicating a characteristic of tuberculous lesion (Figure 1). In mesentery and mesenteric lymph nodes, greenish discharge was observed in the lesions.

### 3.5. Molecular Typing of the Isolates from Goats

**Figure 2:**
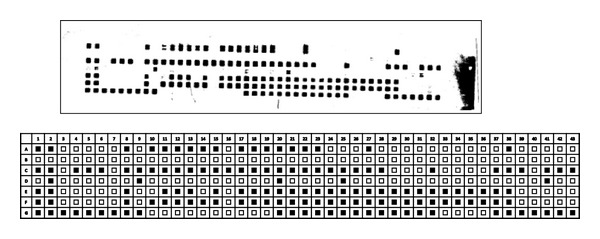
Scanned autorad and schematic representation showing spoligotyping pattern of isolate from the goat with tuberculous lesion caused by *M. tuberculosis*. A: *M. bovis* SB1176 (positive control); B: Qiagen H_2_O (negative control); C: M. tuberculosis (positive control); D–F: sample from other animals, G: SIT149 (isolate from goat). The black rectangles represent presence of spacers, and the white rectangles indicate absence of spacers.

All tissue samples obtained from slaughtered tuberculin reactor goats were positive for mycobacterial growth on LJ culture medium. Further molecular characterization indicated that one of the isolates was human type *Mycobacterium tuberculosis* (SIT149) from goat specimen (Figure 2), and the others were nontuberculosis mycobacteria species. The goat with SIT149 isolate was strong reactor to bovine tuberculin test with high skin induration difference (PPD-B minus PPD-A = 10 mm), and the postmortem examination result showed typical tuberculin lesions in lung, bronchial lymph nodes, caudal mediastinal lymph node, and also on mesenteric lymph nodes while goats from which nontuberculosis mycobacteria species were isolated have showed indurations of skin at both avium and bovine tuberculin injection site. In addition, the pathological lesions observed in postmortem examination were localized in retropharyngeal lymph nodes and mesenteric lymph nodes. 

## 4. Discussion

Little information is available on TB in small ruminants in Ethiopia even though bovine TB is known to be endemic in cattle of Ethiopia [34]. In this study, a prevalence of 0.5% at ≥4 mm cut-off and 3.8% at ≥2 mm cut-off point was recorded in small ruminants in four districts of Afar Pastoral Region of northeastern Ethiopia. The result was in agreement with that of Hiko and Agga [14] who reported 4.2% in goats slaughtered at Mdjo abattoir and with a report by Tafesse and coauthors [17] who recorded a prevalence of 3.1% in goat with single intradermal tuberculin skin test. A recent study carried out on goats and sheep of central Ethiopia using CIDT also showed a low prevalence of tuberculosis (0.41% at 2 mm cut-off point) [16] which might suggests an overall low prevalence of TB in small ruminants in the country. However, our result was different from the result of a previous study done in Hamer pastoral district of southern Ethiopia, which indicated the absence of the disease in 186 goats using CIDT test [21]. This difference might be related, the difference in geographical location of the two studies in which the epidemiology of the disease might vary between these areas. 

The proportion of positive reactors was significantly higher in Dubti district than the other districts which might be related to the husbandry system where small ruminants had higher interaction with cattle in Dubti districts than the other districts, which can favor a potential transmission of mycobacterial species between cattle and goat. Older goat and sheep showed higher proportion of positivity in tuberculin test results which might be related to the fact that older animals have longer duration and repeated chance of exposure to mycobacterial infection with their age. Similar results have been reported by other researchers in cattle [34, 35]. Female animals with more parity number showed higher proportion of positivity in tuberculin test results than in those with lower parity number. This might be related to the age of the animals as animals with high parity number were older in their age which increases their chance of exposure to mycobacterial infection in their longer life time.

Mycobacteriological culture of the tissue lesions from the five tuberculin reactors goats had resulted in the isolation of *Mycobacterium tuberculosis* and nontuberculosis mycobacteria species. In this study, *Mycobacterium tuberculosis* strain SIT149 was isolated from a goat suggesting the possibility of its transmission from human to goat. Similar strain has been isolated in camel from pastoral region in south east of Ethiopia [36]. The SIT149 strain of *Mycobacterium tuberculosis* is a dominant strain in Ethiopia [37], and it was a common isolate in human pulmonary TB patients from the same Afar Pastoral Region indicating that the isolate has been circulating in the area. Afar pastoralists have close contact with goats and sheep and often keep young goats and sheep in their house at night which might be a potential factor for transmission from human patient to animals. Previous studies in cattle of Ethiopia demonstrated that *Mycobacterium tuberculosis* was commonly isolated from tuberculous lesions of cattle in different regions of Ethiopia [38, 39]. 

In sheep, we observed 1.44% prevalence of TB at 2 mm cut-off point and no at 4 mm cut-off point. The result was in agreement with previous studies where sheep TB has been reported both with tuberculin skin test and postmortem examination results [3, 4, 9, 10, 12, 16]. 

In conclusion, this study revealed a moderately low prevalence of TB in goats and sheep of Afar Pastoral Region of Ethiopia. *Mycobacterium tuberculosis *and nontuberculosis mycobacteria were isolated as causative agents of TB in goats of the region. The isolation of the *Mycobacterium tuberculosis* in goat indicates the need for further studies to understand the interspecies transmission dynamics of *Mycobacterium tuberculosis* and the role of small ruminants in the epidemiology of human tuberculosis in pastoralist setting where potential epidemiological risk factors for infection and transmission between livestock and human exist. In addition, the identification of nontuberculosis mycobacteria from tuberculous lesions in goats indicates their importance in the epidemiology of small ruminant TB and further research is needed to identify the species and their public health significance for the pastoralist communities of the region. In general, similar to the previous studies carried out in cattle and camel of pastoral regions of Ethiopia [18, 20–22, 36] which have indicated the endemic nature of tuberculosis in these species, the result of this study also indicated the importance of tuberculosis in small ruminants of Afar Pastoral Region which further emphasizes the need to design a feasible national TB control strategy in livestock of the country. 

## Figures and Tables

**Table 1:** Association of different risk factors to skin test positivity at ≥2 mm cut-off point for small ruminant tuberculosis in Afar Pastoral Region of Ethiopia.

Variables	Number of animals examined	Number of positive (%)	*χ* ^2^	*P* value
Districts				
Chifra	396	20 (5.05)	26.385	0.000^∗^
Dubti	237	22 (9.28)		
Afambo	117	5 (4.27)		
Amibara	1481	39 (2.63)		
Species				
Ovine	347	5 (1.44)	6.460	0.011^∗^
Caprine	1884	81 (4.3)		
Herd size				
≤25	617	32 (5.19)		
11 < *X* ≤ 50	851	32 (3.76)	4.915	0.086
>50	763	22 (2.88)		
Sex				
Male	206	11 (5.34)	1.351	0.245
Female	2025	75 (3.70)		
Age^#^				
≤2	594	18 (3.03)		
2 < *X* < 5	779	36 (4.62)	2.361	0.307
*X* ≥ 5	858	32 (3.73)		
BCS				
Poor	376	13 (3.46)		
Good	1116	48 (4.30)	1.204	0.548
Fat	739	25 (3.38)		
Production system				
Pastoral	2051	80 (3.90)	0.144	0.705
Agropastoral	180	6 (3.33)		
Lactation status				
Kid/lamb	57	2 (3.51)		
Lactating	760	19 (2.50)	1.255	0.534
Nonlactating	354	13 (3.67)		
Reproductive status				
Nonpregnant	857	19 (2.22)	5.342	0.021^∗^
Pregnant	314	15 (4.78)		
Parity number				
<2	260	10 (3.85)		
2 ≤ *X* < 4	304	13 (4.28)	4.415	0.110
*X* ≥ 4	324	5 (1.54)		

^#^ A given age range includes its lower bound and excludes its upper bound; BCS: body condition score; ^∗^statistically significant.

**Table 2:** Multivariable logistic regression analysis of tuberculin reactors with various host-related risk factors at ≥2 mm cut-off point.

Variables	Number of animals examined	Number (%) of positive in CIDT	Crude odds ratio (95% CI)	Adjusted odds ratio (95% CI)
Districts				
Chifra	396	20 (5.05)	1	1
Dubti	237	22 (9.28)	1.92 (1.03–3.61)^∗^	—
Afambo	117	5 (4.27)	0.84 (0.31–2.29)	—
Amibara	1481	39 (2.63)	0.51 (0.29–0.88)^∗^	0.17 (0.05–0.55)^∗^
Species				
Ovine	347	5 (1.44)	1	1
Caprine	1884	81 (4.3)	3.07 (1.24–7.64)^∗^	2.05 (0.42–9.94)
Herd size				
≤25	617	32 (5.19)	1	1
11 < *X* ≤ 50	851	32 (3.76)	0.71 (0.43–1.18)	1.84 (0.52–6.45)
>50	763	22 (2.88)	0.54 (0.31–0.94)^∗^	0.44 (0.14–1.34)
Sex				
Male	206	11 (5.34)	1	1
Female	2025	75 (3.70)	0.68 (0.36–1.31)	0.25 (0.04–1.74)
Age				
≤2	594	18 (3.03)	1	1
2 < *X* < 5	779	36 (4.62)	1.55 (0.87–2.76)	2.16 (0.47–9.89)
*X* ≥ 5	858	32 (3.73)	1.24 (0.69–2.23)	13.79 (2.22–85.55)^∗^
BCS				
Poor	376	13 (3.46)	1	1
Good	1116	48 (4.30)	1.25 (0.67–2.34)	1.90 (0.61–5.88)
Fat	739	25 (3.38)	0.98 (0.49–1.93)	0.75 (0.17–3.28)
Production system				
Pastoral	2051	80 (3.90)	1	1
Agropastoral	180	6 (3.33)	1.18 (0.51–2.74)	—
Lactation status				
Kid/lamb	57	2 (3.51)	1	1
Lactating	760	19 (2.50)	1.05 (0.23–4.77)	0.50(0.06–4.08)
Nonlactating	354	13 (3.67)	0.71 (0.16–3.11)	0.82(0.12–5.79)
Reproductive status				
Nonpregnant	857	19 (2.22)	1	1
Pregnant	314	15 (4.78)	2.21 (1.11–4.41)^∗^	3.43(0.72–16.33)
Parity number				
<2	260	10 (3.85)	1	1
2 ≤ *X* < 4	304	13 (4.28)	1.12 (0.48–2.59)	0.38 (0.09–1.65)
*X* ≥ 4	324	5 (1.54)	0.39 (0.13–1.16)	0.05 (0.01–0.31)^∗^

CI: confidence interval, BCS: body condition scoring, ^∗^statistically significant.

**Table 3:** Multivariable logistic regression analysis of herd TB positivity with selected risk factors at ≥2 mm cut-off point.

Variables	Number of herds examined	Number of positive herds (%)	Crude odds ratio (95% CI)	Adjusted odds ratio (95% CI)
Districts				
Chifra	18	10 (55.6)	1	1
Dubti	13	9 (69.2)	1.8 (0.40–8.07)	2.59 (0.49–13.73)
Afambo	6	2 (33.3)	0.40 (0.06–2.77)	2.70 (0.06–114.64)
Amibara	66	27 (40.9)	0.55 (0.19–1.58)	0.45 (0.14–1.39)
Herd size				
≤25	52	19 (36.5)	1	1
11 < *X* ≤ 50	34	19 (55.9)	2.2 (0.91–5.31)	3.23 (1.21–8.60)
>50	17	10 (58.8)	2.48 (0.81–7.59)	2.48 (0.76–8.09)
Production system				
Agropastoral	8	3 (37.5)	1	1
Pastoral	95	45 (47.4)	1.5 (0.34–6.64)	6.88 (0.28–170.32)

CI: confidence interval.
